# A Retrospective Analysis of Correlations Between Shoulder Impairment and Ultrasound Lymphedema Evaluation in Breast Cancer Patients: Preliminary Results

**DOI:** 10.3390/biomedicines14010104

**Published:** 2026-01-05

**Authors:** Gianpaolo Ronconi, Rossella Calciano, Alberto Cutaia, Mariantonietta Ariani, Elisabetta Lama, Lucia Forastiere, Sara Corsini, Paola Emilia Ferrara

**Affiliations:** 1 Department of Geriatrics, Orthopedics and Rheumatology, Fondazione Policlinico Universitario “Agostino Gemelli” IRCCS, Largo A. Gemelli 8, 00168 Rome, Italy; gianpaolo.ronconi@policlinicogemelli.it (G.R.); rossella.calciano@guest.policlinicogemelli.it (R.C.); paolaemilia.ferrara@policlinicogemelli.it (P.E.F.); 2 Department of Geriatric and Orthopedic Sciences, Università Cattolica del Sacro Cuore, L.go F. Vito 1, 00168 Rome, Italy; mariantoniettaariani11@gmail.com (M.A.); elisabetta.lama@outlook.it (E.L.); lucia.forastiere@guest.policlinicogemelli.it (L.F.); sara.corsini@guest.policlinicogemelli.it (S.C.)

**Keywords:** lymphedema, breast cancer, rehabilitation, shoulder impairment, range of motion, ultrasound

## Abstract

**Background/Objective:** Breast cancer-related lymphedema (BCRL) is a frequent postoperative complication associated with shoulder functional impairment. Early diagnosis and comprehensive assessment are recommended, yet the literature shows heterogeneity regarding instrumental tools and the role of soft tissue ultrasound is not yet standardized. The aim of this retrospective observational study is to evaluate correlations between shoulder impairment and lymphedema in breast cancer patients. **Methods:** Medical records of 27 outpatient women after breast cancer surgery (mean age ± SD: 55.10 ± 9.58) were evaluated. Clinical variables included anamnestic data regarding surgery and oncology treatments, limb circumferences, passive shoulder range of motion (PROM), axillary web syndrome (AWS) and BMI. Assessment tools included DASH, ECOG, and VAS. Millimetric ultrasound measurements of the dermo-epidermal complex and subcutaneous tissue, at standardized sites, were performed to study limb lymphedema. **Results:** Lymphedema was found in 35.7% of patients. They showed higher rates of lymphadenectomy, AWS, higher BMI, limited shoulder flexion (*p* = 0.002) and abduction (*p* = 0.004), and higher DASH scores (31.99 ± 15.70 vs. 26.16 ± 17.8) compared with patients without lymphedema. There was a preliminary correspondence between circumferential and ultrasound measurement sites of patients’ lymphedema limbs. **Conclusions:** Ultrasound evaluation associated with functional assessment may support the early diagnosis of shoulder impairment and higher limb lymphedema risk to improve rehabilitation treatments in patients after breast cancer surgery.

## 1. Introduction

Breast cancer is the most prevalent malignancy among women worldwide [1] and currently the 5-year survival rate has surpassed 90% [2].

Considering the development and evolution of oncological treatments, substantial innovations have been introduced in recent years in treatment and diagnosis. Future research aims to set therapies in order to improve survival, and new tools like ultrasound are supportive assessment instruments that become essential in rehabilitation as treatment complexity increases [3].

Lymphedema affects 20–40% of patients after breast cancer surgery and oncological treatments [1]. According to the 2023 ISL Consensus, it is defined as a clinical manifestation of lymphatic system insufficiency and impaired lymph transport, resulting in the accumulation of protein-rich interstitial fluid in tissues. It may be primary (congenital) or secondary (acquired, e.g., post-surgical or post-radiotherapy) and can lead to chronic tissue changes if untreated [4].

Clinically, lymphedema presents as gradually increasing swelling in the arm, shoulder and neck, typically associated with sensations of discomfort, tightness, and heaviness, which may lead to reduced function and a lower quality of life [5]. It is characterized by chronic inflammation and results in the presence of fibrous connective tissue and muscle fibrosis [5,6,7,8,9].

Breast cancer-related lymphedema represents an important condition after surgery and affects the upper limbs, with a reduction in quality of life and a major impact on physical and social burden. It interferes with the patient’s daily life activities and general wellbeing [5]. In the current literature, the assessment of breast cancer-related lymphedema and upper limb impairment is heterogeneous, relying on various clinical tools. Prevention of lymphedema is crucial, as well as early limb functional evaluation. Timely intervention can limit disease progression, reduce long-term functional impairment, and improve quality of life. Furthermore, early rehabilitative assessment and a standardized follow-up can improve clinical outcome, with a reduction in health and socioeconomic costs [6].

Although nowadays there is an improvement in diagnostic strategies and treatment with early detection of this condition, lymphedema frequently remains undiagnosed until the edema becomes apparent. Delays in diagnosis result in delayed therapeutic intervention and patient care, and consequently a reduction in the efficacy of treatment. Early detection enables the start of rehabilitation via Complete Decongestive Therapy (CDT), a personalized approach tailored to the severity of this condition that includes compression therapy, manual lymphatic drainage, specific exercises, skin care, and patient education [10,11]. Evaluating the severity of breast cancer-related lymphedema (BCRL) and the impairment involves the use of multiple clinical assessment tools; however, there is a lack of a standardized method of assessment.

The use of ultrasound (US) has demonstrated considerable potential in both diagnosing lymphedema and determining its severity. Recent studies have highlighted its value in the detection and longitudinal monitoring of lymphedema progression [12,13,14].

US evaluation matters for rehabilitation and survivorship rather than being merely a technical add-on. It is particularly advantageous because it allows for detailed visualization of various tissue layers: skin, subcutaneous tissue, and muscle. This technique thereby facilitates the identification of localized fluid accumulation and alterations in fibroadipose tissue within the affected limb [15].

It is a quick, non-invasive and well-tolerated instrument that is easily applicable in clinical practice as well as available in rehabilitation. Nevertheless, significant uncertainty remains regarding the impact of US on the prognosis of lymphedema and on outcomes reported by patients. As shown by Mander et al. [16], US remains poorly utilised in the routine evaluation of lymphedema, despite its potential to characterize tissue alterations. A study conducted on 287 patients supports its value in improving the understanding of lymphatic pathophysiology and guiding more targeted therapeutic strategies.

Furthermore, lymphedema is related to a reduction in shoulder range of movement with upper limb impairment due to the decrease in scapular upward rotation and posterior tilt [17,18,19].

Moreover, the overall effects of breast cancer treatment on upper body morbidity should be recognized as a key clinical consideration and proactively addressed to enhance patient outcomes [19].

Upper limb dysfunction occurs in up to 82% of breast cancer survivors, restricting daily activities such as reaching and self-care and substantially diminishing quality of life. Recent studies have shown that these survivors often exhibit altered shoulder muscle coordination, characterized by heightened activation of the upper trapezius and deltoid muscles, along with delayed engagement of stabilizing muscles during arm movements [20].

However, in the literature, ultrasound assessment protocols for upper limb lymphedema after breast cancer are extremely heterogeneous, both in terms of reference points and protocols used, as well as the layers analyzed. Some studies assess only the skin and subcutaneous tissue, while others include the skin, subcutaneous layer, and fascia. This heterogeneity makes it difficult to establish a standardized protocol for the assessment, as well as a standardized correlation with shoulder impairment [4,5,21,22].

Recently, authors have investigated the association between lymphedema severity and shoulder function using both objective assessments (such as range of motion and muscle strength) and subjective measures (such as the DASH questionnaire) [23].

Furthermore, early patient evaluation requires timely diagnosis of both lymphedema and upper limb dysfunction to optimize recovery and functional outcomes [17,18,19,20].

The aim of this study is to evaluate, through a retrospective analysis of outpatient women, lymphedema and shoulder impairment in breast cancer patients.

## 2. Materials and Methods

This is an observational retrospective study. The medical records of 27 outpatient women who had undergone breast cancer surgery (mean age ± SD: 55.10 ± 9.58) were retrospectively reviewed after obtaining informed consent. All patients were evaluated at the Physical Medicine and Rehabilitation outpatient clinic of the Policlinico Gemelli Foundation (L.go A. Gemelli 8, 00168 Rome, Italy) in Rome between January and May 2025. The inclusion criteria for this study were a confirmed diagnosis of breast cancer and subsequent surgical treatment, a minimum age of 18 years, and signed consent for the study.

Exclusion criteria were primary lymphedema and refusal to sign informed consent. Patients were evaluated using routine clinical and practice with a standardized core set which included the following validated tools: disabilities of the Arm, Shoulder, and Hand (DASH), the Eastern Cooperative Oncology Group (ECOG) scale, the Visual analogical Pain Scale (VAS), the Goniometric Shoulder passive range of motion (PROM), and limb circumferential measurements (starting from the wrist crease, every 5 cm up to the axilla and up to the hand), where a difference of at least two cm in two points between the limbs was indicative of lymphedema [21].

All patients underwent evaluation using high-resolution ultrasound as per usual clinical practice (Esaote^®^ ultrasound system equipped with a 6–16 MHz multifrequency linear probe) examining the millimetric ultrasound measurements of the dermo-epidermal complex across 17 predefined limb regions according to the Mander protocol [16]. We only assessed dermo-epidermal thickness and not qualitative patterns, which are operator-dependent. An expert operator performed the ultrasound examination.

As this study is retrospective and the ultrasound protocol was part of routine clinical practice, formal ethical approval was considered unnecessary. All patients provided consent for the anonymized use of their data for research purposes.

### Statistical Analysis

Statistical analysis was performed using SPSS version 14. Descriptive data were reported for ordinal and nominal data, while mean and standard deviation were reported for continuous-interval data. The clinical, demographic, and laboratory characteristics of the sample are summarized using descriptive statistics. Quantitative variables are reported as mean and standard deviation if normally distributed. Qualitative variables are reported as absolute frequency and percentage. The normality of continuous variables was verified using the Shapiro–Wilk test. A mixed-effects model was employed to assess temporal variations in the scores, accounting for group effects and potential confounding variables. A 95% confidence level was used, and statistical significance was set at *p* ≤ 0.05. Data comparisons between patients with and without lymphedema were performed using Student’s *t*-test for independent variables and the χ test for categorical variables.

Given the retrospective nature of the study and the use of anonymized data collected as a part of clinical practice, Ethics Committee approval was not deemed necessary. The ultrasound protocol was used as in normal clinical practice and all patients signed informed consent for the anonymous use of their data.

## 3. Results

Table 1 shows the characteristics of the sample, clinical data and outcome measures. A total of 27 patient records were included in the study (10 Lymphedema group, LI, and 17 No Lymphedema group, NoLI). There was a mean age of 55.3 ± 11.1 years and a mean BMI of 24.3 ± 3.5. Surgical interventions included quadrantectomy (46.4%), unilateral mastectomy (42.9%), and bilateral mastectomy (10.7%). Surgery was performed on the right side in 39.3% and on the left side in 57.1% of patients. Half of the participants underwent reconstructive surgery. Regarding lymph node management, 96.4% underwent sentinel lymph node biopsy and 92.9% underwent lymphadenectomy. Postoperative seroma occurred in 17.9% of cases. Comorbidities were present in 14.3% of patients, with 46.4% having more than one. Treatment modalities included neoadjuvant chemotherapy in 32.1% of patients and adjuvant chemotherapy in 32.2% (21.5% within 0–3 months, 3.6% within 6–12 months, and 7.1% more than 2 years prior to assessment). Radiotherapy was administered to 42.9% (0–3 months), 25% (6–12 months), and 10.7% (>2 years), with 21.4% not receiving radiotherapy. Biological therapy and hormone therapy were used in 28.6% and 88.9% of patients, respectively.

Regarding lymphedema and related complications, 35.7% had lymphedema, 17.9% underwent lymphatic drainage, 17.9% had axillary web syndrome, 10.7% tested positive for the Stemmer sign, and 7.1% had a fovea.

Functional outcomes included a mean DASH score of 28.2 ± 17.1, ECOG performance status of 0.89 ± 0.68, and VAS pain score of 2.21 ± 2.69.

Table 2 compares the two groups, LI and NoLI. Patients with lymphedema had a higher incidence of lymphadenectomy (100% vs. 89%, *p* = 0.003) and seroma formation (40% vs. 5.6%, *p* < 0.001). Differences were also observed in neoadjuvant chemotherapy (20% vs. 38.9%, *p* = 0.032) and the timing of adjuvant chemotherapy (*p* < 0.001). Lymphedema was associated with a higher prevalence of axillary web syndrome (40% vs. 5.6%, *p* < 0.001), positive Stemmer sign (30% vs. 0%, *p* < 0.001), and fovea (20% vs. 0%, *p* < 0.001). The use of compression garments was less frequent in the lymphedema group (60% vs. 83.3%, *p* = 0.018). Shoulder range of motion was reduced in lymphedema patients for flexion (*p* = 0.022) and abduction (*p* = 0.004).

**Figure 1:**
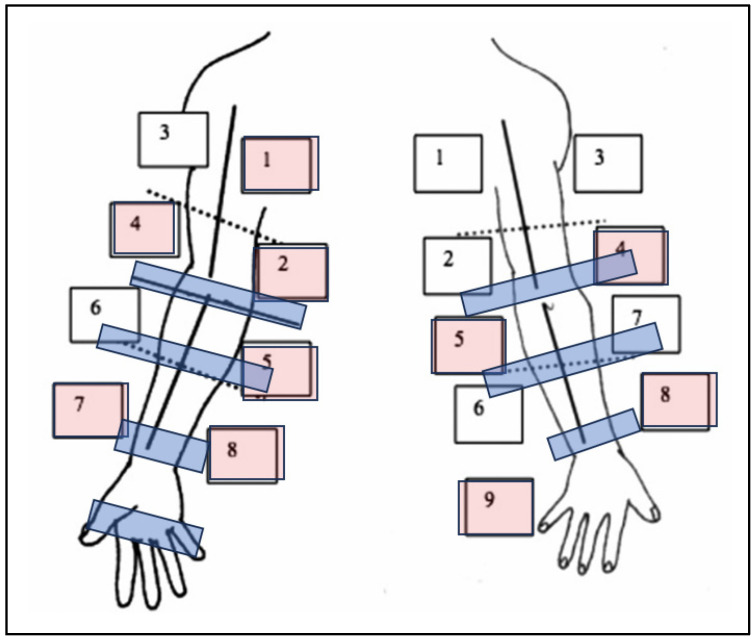
Red-coded ultrasound indicates significant millimetric points of dermo-epidermal layer and blue-coded regions correspond to significant millimetric circumferences.

Table 3 shows circumferential arm measurements. Significant differences in limb measurements were observed at the left elbow (*p* = 0.018), left forearm (*p* = 0.046), right wrist (*p* = 0.049), left wrist (*p* = 0.033), and right arm (*p* = 0.039). Ultrasound dermo-epidermal complex thickness was greater in lymphedema patients at right anterior points 4 (*p* = 0.047), 5 (*p* = 0.002), and 8 (*p* = 0.002), and right posterior points 7 (*p* = 0.005), 8 (*p* = 0.019), and 9 (*p* = 0.013) (Figure 1).

## 4. Discussion

The aim of this observational retrospective study was to evaluate lymphedema and shoulder impairment in breast cancer patients.

Our preliminary findings are consistent with the existing literature and confirm the presence of known lymphedema risk factors, including higher mean BMI, more extensive lymph node dissection, and a greater incidence of web axillar syndrome among patients with lymphedema [21].

In the literature, evaluations of breast cancer-related lymphedema and post-surgical limb impairment vary widely, with studies using a range of different clinical assessment tools [16]. Many studies indicate that, despite current monitoring strategies focusing on early detection, BCRL frequently goes unnoticed until significant swelling develops. This delay in diagnosis may limit the effectiveness of early treatments and negatively affect patients’ functional abilities and overall quality of life [6].

Patients with lymphedema presented significant shoulder impairment, with a reduction in the range of motion of the arms in flexion and abduction. These results align with Mohamed et al., 2024 [17], establishing that the severity of lymphedema is correlated with gradual reductions in shoulder mobility, strength, and overall function. Patients with severe lymphedema exhibited significantly limited shoulder flexion, abduction, rotation, and extension. These findings indicate that impaired shoulder joint control contributes to difficulties in arm elevation and daily activities.

Tugral et al., in a recent research article (2025), investigated factors affecting upper extremity function in breast cancer survivors. Among 100 participants, handgrip strength, fatigue and self-reported upper limb function (DASH) were assessed. Results showed that right-hand strength was higher than left-hand strength. In particular, fatigue and DASH scores were moderately correlated (*p* < 0.001) [19].

Ultrasound is a non-invasive and increasingly adopted technique that shows strong potential for diagnosing lymphedema and assessing its severity. Although standardized assessment tools, methodology and protocol for ultrasound evaluation of lymphedema are still lacking, high-frequency ultrasound is already becoming part of standardized assessment in some centers. A systematic review conducted by de Rezende et al., 2023, underlined that US could be a diagnostic tool and could assess soft tissue characteristics and tissue compliance, aiding in both diagnosis and management [24].

Klarić-Kukuz proposed that ultrasound be typically performed bilaterally at five standardized sites for lymphedema measurement [21]. Instead, Yuk D. et al. assessed lymphedema sonographic evaluation considering four points of the upper arm and forearm, comparing both sides [22]. Our evaluation was conducted analyzing sites and thicknesses as shown in Mander et al.’s 2019 study by the same operator [16]. This protocol seemed to offer greater accuracy, enabling comparison with circumferential measurements and providing a more precise evaluation of both lymphedema severity and appropriate rehabilitation approaches. Our study differs in that we analyzed only the dermo-epidermal thickness, rather than qualitative features, which are more affected by operator dependency.

Pirri et al. (2024) evaluated through high-resolution ultrasound 14 patients with stage II upper-limb lymphedema. This analysis revealed region-specific increases in cutis and subcutis thickness and altered subcutaneous echogenicity, while fascial thickness remained unchanged. These findings support targeted rehabilitation and more precise lymphedema management based on affected areas [5].

Based on this, a tailored rehabilitation plan can be developed, in which some areas are affected earlier than others. Furthermore, ultrasound may provide early detection of dermal–epidermal changes.

This study has several limitations. First, the relatively small sample size reflects the preliminary and exploratory nature of the study and was mainly determined by the strict inclusion criteria and the limited availability of complete clinical and ultrasound data within the study period at our institution. Owing to the small sample size, formal justification of the sample size and calculation of confidence intervals were not performed. As a result, the precision of the estimated effects cannot be fully quantified, which may affect the accuracy and internal validity of the findings. Second, the retrospective design and single-center setting may introduce selection bias and limit the generalizability of the results to broader populations and different clinical settings. In addition, ultrasound is an operator-dependent technique requiring adequate training and experience, which may further affect reproducibility. Overall, these limitations restrict both the internal and external validity of the study, and the findings should therefore be interpreted with caution and considered hypothesis-generating rather than definitive. Future prospective, multicenter studies with larger sample sizes, formal sample size calculations, standardized ultrasound protocols, and long-term follow-up are warranted to confirm these results and improve their generalizability.

## 5. Conclusions

Our findings suggest that ultrasound could be an important tool in the management of breast cancer-related lymphedema and shoulder impairment. The integration of a structured assessment that includes ultrasound demonstrated its utility in the early identification and treatment of upper-limb impairment and lymphedema, as well as in monitoring the patient’s condition during follow-up.

Due to the limitations of this analysis, it is essential to standardize evaluation procedures and ensure the systematic use of ultrasound. Its incorporation into routine management pathways supports the planning of individualized rehabilitation strategies and allows for the reliable evaluation of treatment responses, including the effectiveness of complex decongestive therapy.

## Figures and Tables

**Table 1:** Patient clinical data and outcome measures.

Variable	Value
Age, years	55.28 ± 11.12
Body mass index, kg/m^2^	24.30 ± 3.52
Type of breast surgery, *n* (%)	
—Quadrantectomy	46.4
—Unilateral mastectomy	42.9
—Bilateral mastectomy	10.7
Side of surgery, *n* (%)	
—Right	39.3
—Left	57.1
Plastic surgery, *n* (%)	
—Yes	50.0
—No	50.0
Lymph node procedures, *n* (%)	
—Sentinel lymph node biopsy	96.4
—Axillary lymphadenectomy	92.9
Post—surgical complications, *n* (%)	
—Seroma	17.9
—Axillary web syndrome	17.9
Comorbidities, *n* (%)	
—None	39.3
—One	14.3
—>1	46.4
Neoadjuvant chemotherapy, *n* (%)	
—Yes	32.1
—No	67.9
Adjuvant chemotherapy, *n* (%)	
—0–3 months before evaluation	21.5
—6–12 months before evaluation	3.6
—>2 years before evaluation	7.1
—No	67.9
Radiotherapy, *n* (%)	
—0–3 months before evaluation	42.9
—6–12 months before evaluation	25.0
—>2 years before evaluation	10.7
—No	21.4
Biological therapy, *n* (%)	
—Yes	28.6
—No	71.4
Hormone therapy, *n* (%)	
—Yes	88.9
—No	11.1
Lymphedema—related findings, *n* (%)	
—Lymphedema	35.7
—Manual lymphatic drainage	17.9
—Positive Stemmer sign	10.7
—Positive fovea sign	7.1
Outcome measures (mean ± SD)	
—DASH score	28.24 ± 17.08
—ECOG performance status	0.89 ± 0.68
—VAS pain score	2.21 ± 2.69

Abbreviations: BMI, body mass index; DASH, Disabilities of the Arm, Shoulder and Hand; ECOG, Eastern Cooperative Oncology Group; VAS, visual analogue scale. Data are presented as mean ± standard deviation or *n* (%), as appropriate.

**Table 2:** Comparison between patients with and without lymphedema.

Variable	Lymphedema Group (*n* = 10)	No Lymphedema Group (*n* = 17)	Measure	*p*-Value
Age, years	53.10 ± 9.58	56.50 ± 12.28	Mean ± SD	
Body mass index, kg/m^2^	25.20 ± 4.52	23.64 ± 3.32	Mean ± SD	
Type of breast surgery, *n* (%)				
—Quadrantectomy	60	33	*n* (%)	
—Unilateral mastectomy	30	55	*n* (%)	
—Bilateral mastectomy	10	12	*n* (%)	
Side of surgery, *n* (%)				
—Right	40	38.9	*n* (%)	
—Left	60	55.6	*n* (%)	
Sentinel lymph node biopsy, *n* (%)				
—Yes	90	94.5	*n* (%)	
—No	10	5.6	*n* (%)	
Axillary lymphadenectomy, *n* (%)				0.003
—Yes	100	89	*n* (%)	
—No	–	11	*n* (%)	
Seroma, *n* (%)				0.000
—Yes	40	5.6	*n* (%)	
—No	60	94.4	*n* (%)	
Comorbidities, *n* (%)				
—None	30	44.4	*n* (%)	
—One	30	5.6	*n* (%)	
—>1	40	50	*n* (%)	
Neoadjuvant chemotherapy, *n* (%)				0.032
—Yes	20	38.9	*n* (%)	
—No	80	61.1	*n* (%)	
Adjuvant chemotherapy, *n* (%)				0.000
—0–3 months	20	16.7	*n* (%)	
—6–12 months	10	5.6	*n* (%)	
—>2 years	20	–	*n* (%)	
—No	50	77.8	*n* (%)	
Radiotherapy, *n* (%)				
—0–3 months	30	44.5	*n* (%)	
—6–12 months	30	27.8	*n* (%)	
—>2 years	20	5.6	*n* (%)	
—No	20	22.2	*n* (%)	
Biological therapy, *n* (%)				
—Yes	20	33.3	*n* (%)	
—No	80	66.7	*n* (%)	
Hormone therapy, *n* (%)				
—Yes	90	88.9	*n* (%)	
—No	10	11.1	*n* (%)	
Lymphatic drainage, *n* (%)				
—Yes	40	5.6	*n* (%)	
—No	60	94.5	*n* (%)	
Axillary web syndrome, *n* (%)				0.000
—Yes	40	5.6	*n* (%)	
—No	60	94.4	*n* (%)	
Stemmer sign, *n* (%)				0.000
—Positive	30	–	*n* (%)	
—Negative	70	100	*n* (%)	
Fovea sign, *n* (%)				0.000
—Positive	20	–	*n* (%)	
—Negative	80	100	*n* (%)	
Compression garments, *n* (%)				0.018
—Yes	60	83.3	*n* (%)	
—No	40	16.7	*n* (%)	
Shoulder PROM—Flexion, *n* (%)				0.022
—120–180	70	66.7	*n* (%)	
—60–120	20	11.1	*n* (%)	
—0–60	10	22.2	*n* (%)	
Shoulder PROM—Abduction, *n* (%)				0.004
—120–180	80	66.6	*n* (%)	
—60–120	20	16.7	*n* (%)	
—0–60	–	16.7	*n* (%)	
DASH score	31.99 ± 15.70	26.16 ± 17.89	Mean ± SD	
ECOG performance status	1.10 ± 0.56	0.77 ± 0.73	Mean ± SD	
VAS pain score	2.50 ± 2.95	2.05 ± 2.62	Mean ± SD	

Data are presented as mean ± standard deviation or *n* (%). *p*-values refer to between-group comparisons. PROM: passive range of motion.

**Table 3:** Significant circumferential measurements and millimetric ultrasounds of dermo-epidermal complex between patients with and without lymphedema.

Measurement Site	Lymphedema Group (*n* = 10) Mean ± SD	No Lymphedema Group (*n* = 17) Mean ± SD	*p*-Value
Circumferential measurements (cm)			
Right elbow	25.30 ± 2.82	23.47 ± 2.02	0.057
Left elbow	26.10 ± 3.75	23.36 ± 2.02	0.018
Right forearm	22.91 ± 2.93	20.27 ± 5.18	0.098
Left forearm	24.25 ± 4.04	20.44 ± 5.30	0.046
Right wrist	16.40 ± 1.61	14.25 ± 3.82	0.049
Left wrist	16.90 ± 2.18	14.30 ± 3.89	0.033
Left hand	19.80 ± 2.79	17.44 ± 4.17	0.087
Right arm (index)	1.20 ± 0.42	1.16 ± 0.38	0.039
Ultrasound dermal–epidermal thickness (mm)			
Right anterior—point 2	1.76 ± 0.42	1.47 ± 0.50	0.092
Right anterior—point 4	2.80 ± 1.91	1.82 ± 0.46	0.047
Right anterior—point 5	1.98 ± 0.54	1.41 ± 0.33	0.002
Right anterior—point 7	1.91 ± 0.49	1.55 ± 0.45	0.077
Right anterior—point 8	1.98 ± 0.52	1.45 ± 0.25	0.002
Left anterior—point 1	1.75 ± 0.64	1.41 ± 0.31	0.071
Right posterior—point 5	2.46 ± 2.01	1.60 ± 0.23	0.081
Right posterior—point 7	1.96 ± 0.59	1.46 ± 0.26	0.005
Right posterior—point 8	2.05 ± 0.81	1.53 ± 0.26	0.019
Right posterior—point 9	1.93 ± 0.88	1.28 ± 0.41	0.013

Data are presented as mean ± standard deviation. *p*-values refer to between-group comparisons. Circumferential measurements are expressed in centimeters (cm) and ultrasound dermal–epidermal thickness in millimeters (mm).

## Data Availability

The datasets generated and/or analyzed during the current study are available from the corresponding author upon reasonable request.
